# Action of Medicine

**Published:** 1878-04

**Authors:** 


*The Action of Medicine.* By Isaac Ott, A. M., M. D. Formerly Demonstrator of Experimental Physiology, University of Pennsylvania. With twenty-two illustrations. Philadelphia: Lindsay & Blakiston. 1877. Buffalo: T. H. Butler. pp. 164. Price, $2.00.

The wide spread of skepticism in medical circles with regard to the possible beneficient power of drugs which has touched the profession like a blight promises to be of use in stimulating investigation, and is already leading to greater exactness of knowledge.

Dr. Ott has prepared this little volume as a text book for classes in practical physiology but it cannot fail to be valued by thinkers and workers who have neither time nor opportunity for extensive vivisection. " How to study the physiological action of medicines." "Action on the nervous system" and "Action on the circulatory apparatus" are treated in different chapters." Suggestions as to animals used, plates of apparatus and modes of procedure are given.

The fourth and closing chapter is devoted exclusively to medicines, and contains most of what is known of the action of some thirty or forty powerful agents, arranged under the usual heads; "Action on lower animals, on man, and in disease." It is not a book calculated to please the cursory reader and must be studied to be appreciated. It is a credit to the profession that the want of a text book of this sort has been felt, and not less creditable, that the want has called forth a response from one careful worker, clear thinker, and judicious teacher. M. B. M.

